# Machine Learning Applied to COVID-19: A Review of the Initial Pandemic Period

**DOI:** 10.1007/s44196-023-00236-3

**Published:** 2023-05-07

**Authors:** Leandro Y. Mano, Alesson M. Torres, Andres Giraldo Morales, Carla Cristina P. Cruz, Fabio H. Cardoso, Sarah Hannah Alves, Cristiane O. Faria, Regina Lanzillotti, Renato Cerceau, Rosa Maria E. M. da Costa, Karla Figueiredo, Vera Maria B. Werneck

**Affiliations:** Department of Informatics and Computer Science, Rio de Janeiro State University, Rua São Francisco Xavier 524, Rio de Janeiro, Rio de Janeiro, 20550-900 Brazil

**Keywords:** COVID-19, Machine learning, Image data, Clinical and laboratorial data, Diagnosis/outcome

## Abstract

Diagnostic and decision-making processes in the 2019 Coronavirus treatment have combined new standards using patient chest images, clinical and laboratory data. This work presents a systematic review aimed at studying the Artificial Intelligence (AI) approaches to the patients’ diagnosis or evolution with Coronavirus 2019. Five electronic databases were searched, from December 2019 to October 2020, considering the beginning of the pandemic when there was no vaccine influencing the exploration of Artificial Intelligence-based techniques. The first search collected 839 papers. Next, the abstracts were reviewed, and 138 remained after the inclusion/exclusion criteria was performed. After thorough reading and review by a second group of reviewers, 64 met the study objectives. These papers were carefully analyzed to identify the AI techniques used to interpret the images, clinical and laboratory data, considering a distribution regarding two variables: (i) diagnosis or outcome and (ii) the type of data: clinical, laboratory, or imaging (chest computed tomography, chest X-ray, or ultrasound). The data type most used was chest CT scans, followed by chest X-ray. The chest CT scan was the only data type that was used for diagnosis, outcome, or both. A few works combine Clinical and Laboratory data, and the most used laboratory tests were C-reactive protein. AI techniques have been increasingly explored in medical image annotation to overcome the need for specialized manual work. In this context, 25 machine learning (ML) techniques with a highest frequency of usage were identified, ranging from the most classic ones, such as Logistic Regression, to the most current ones, such as those that explore Deep Learning. Most imaging works explored convolutional neural networks (CNN), such as VGG and Resnet. Then transfer learning which stands out among the techniques related to deep learning has the second highest frequency of use. In general, classification tasks adopted two or three datasets. COVID-19 related data is present in all papers, while pneumonia is the most common non-COVID-19 class among them.

## Introduction

In December 2019, China announced the emergence of a new coronavirus infection in humans in Wuhan (China). The disease spread rapidly worldwide and was declared as a pandemic with significant morbidity and mortality by the World Health Organization (WHO) [1, 2] in March 2020. A ribonucleic acid (RNA) virus named severe acute respiratory syndrome coronavirus 2 (SARS-CoV-2) causes coronavirus disease 2019 (COVID-19). The current pandemic succeeds the significant occurrences of severe acute respiratory syndrome (SARS, caused by SARS-CoV-1 virus) in 2002–2004 and middle east respiratory syndrome (MERS, caused by MERS-COV virus) in 2012 [3]. According to WHO,^1^ until February, 2023, morbidity had reached more than 757 million cases and almost 6.850 million deaths, characterizing itself as a principal public health problem to be tackled with the help of applied science.

After the first day of contamination, common clinical manifestations such as fever, cough, fatigue, sore throat, headache and shortness of breath may appear. There is high transmissibility of the virus. Droplets, aerosols or contact with contaminated surfaces can spread the infection. In addition, there is a possibility of transmission by asymptomatic people or even before the onset of symptoms. Some groups of patients are more likely to progress to pneumonia, respiratory failure and death. Treatment is essentially supportive for symptomatic patients [4].

According to the World Health Organization, of the more than 100 vaccines proposed for COVID-19, 10 candidate vaccines had reached the human trial phase (clinical trials), as presented in the World Report of Lacet on June 6, 2020. Various technologies and compounds are in use to produce the vaccines, such as a virus (Inactivated and Weakened), viral vector (replicating and non-replicating), nucleic acid (DNA (deoxyribonucleic acid) and RNA) and protein-based vaccine (protein subunit and virus-like particles) [5].

In addition, as a way to control the spread of the virus, personal and environmental restrictions have been implemented in several countries, such as the use of masks, social distancing, closing of commercial establishments, schools, and universities [6]. The first impacts generated many concerns due to the high rate of contamination with a high number of deaths. However, the emergence of new strains and complications after the disease are still a matter of concern. This scenario may continue until widespread adoption of the COVID-19 vaccine.

The COVID-19 pandemic has mobilized governments, universities, and research centers around the world. However, now the scenario is still worrying due to the high rate of virus variants and because the disease complications are related to increasing long term sequelae. This scenario may continue until the full adoption of the vaccine against COVID-19.

Thus, this COVID-19 scenario has brought together specialists and researchers from different areas in initiatives that can help combat the disease. Above all, artificial intelligence (AI) has directed efforts to develop solutions to the crisis. In different contexts, similar resources have been used such as diagnosis and treatment, prediction of the disease spreading, development of new drugs and vaccines, management of hospital beds and supplies, identification of crowds of people, in addition to economic analysis due to social isolation and fighting fake news [7].

In the efforts against COVID-19, AI can play an essential role in the diagnosis and outcome of the disease. When addressing AI in this context, machine learning (ML) methods stand out and have been applied to classify different medical image modalities, using a variety of attributes, for several diseases and tools, such as computer-aided diagnosis/detection and radiomics [8-10]. The development of a ML method involves creating a training function for a dataset, making use of a logical inference mechanism [11]. Indeed, such an approach can be used in different ways in the analysis of medical images or clinical data of the patient, for example, in the area of radiology and diagnostic imaging, they have been mainly applied in computer-aided diagnosis/detection, content-based image retrieval and radiomics/radiogenomics [10, 11]. The trained algorithms can make predictions with adequate speed and accuracy. The purpose is to identify non-visible patterns, indications, among other events, with the probability of the disease’s progression or complication [12].

This systematic review aimed to study the approaches of AI applied to the diagnosis or outcome of patients with COVID-19 published until the beginning of the pandemic when there was no vaccine. This period aimed at identifying patterns that mapped relationships between clinical data and images with diagnoses and outcomes, with a high volume of works involving diagnosis. Furthermore, AI classification methodologies and models were identified.

The Covid-19 epidemic and the spread of AI/ML have increased studies on the exploration of AI/ML techniques applied to various dimensions of the epidemic: drugs and vaccines development [13], models of disease advance [14], use of images [15] and clinical data [16] for disease diagnosis and outcome.

In this context, some literature reviews were carried out and identified the use of AI/ML techniques to model, simulate and project COVID-19. The review described by Dogan et al. [17] pointed out that the convolutional neural network (CNN) was the most used network model to diagnose COVID-19 from X-ray exams. ResNet was the most used network for classification. Logistic regression was the most used model for prediction purposes. However, this work does not accurately determine the time window in which the works were published. Abd-Alrazaq et al. [18] carried out a review considering works published from Dec 2019 to April 2020. The limitation of this paper is that as the pandemic was at the beginning, the review considered many preprints that have not yet been evaluated by experts. This paper identified works carried out with clinical, laboratory and image databases, in which the most used model was CNN, followed by support vector machines (SVM) and recurrent neural networks (RNN). In the review developed by Islam et al. [19], 49 studies were identified. They explored AI techniques to diagnose, analyze and predict risks and project the progress of the epidemic, among other less frequent ones. Among these works, 10 used images to mostly apply the CNN model. Their work presented some still incipient papers which analyzed the possibilities of AI in the identification, dissemination, and control of the disease via vaccination.

Our proposal complements some of these works, which considered reviews carried out at the beginning of the pandemic, when there was not much knowledge about the use of AI/ML techniques with the COVID-19. Abd-Alrazaq et al. [18] considered works until April 2020; the paper by Goel [14] was published in July 2020 and Islam et al. [19], whose research was up to August 2020, considered early work on the use of AI techniques on COVID-19 data.

The main contributions of this systematic review are: (i) identification of studies that explored AI techniques considering imaging, laboratory, and clinical data to generate diagnoses or outcomes related to COVID-19 when the vaccine had not yet been distributed; (ii) present results, with several data crossings, carried out by a large team that was able to perform three rounds of reviews to identify the most used AI and ML techniques in the context of the pandemic; (iii) describes all datasets associated with each work; (iv) an analysis of the adoption of AI methods related to the attributes of data used in the studies; and (v) a discussion of the most used AI/ML techniques associated with each data type.

This review is organized as follows: Sect. 2 presents the adopted methodology considering PRISMA [20] criteria, describing the search strings, databases, and studies selection strategies. It describes the collection phase, presents a flowchart that summarizes results from the application of PRISMA steps, a summary of an analysis of the selected studies, a Sankey diagram with the distribution of papers concerning their data types and purpose, and a discussion about the terms that appeared in the studies. In Sect. 3, we analyze the major AI approaches adopted in the studies relating them to the data type: images, laboratory and clinical. Section 4 discusses the results, presenting the frequency of the machine learning techniques used in the papers. Section 5 describes some limitations of this study. Finally, Sect. 6 concludes, stressing some perceptions about the adoption of AI techniques and some challengers.

## Methodology

A systematic literature review (SLR) identifies, analyses, and interprets available evidence related to specific research. The methodology of this systematic review was conducted according to preferred reporting items for systematic reviews and meta-analyses (PRISMA) criteria [20]. The process defined an eligibility criterion that identifies the review goals, the inclusion, and the exclusion criteria applied. This section also identifies the databases used, the search strategy, the selection steps, and the study characteristics that show the papers selected in this SLR.

### Eligibility Criteria

This systematic review included: (1) studies regarding the use of the AI approach for the diagnosis or outcome of patients with COVID-19, either by imaging (chest X-Ray, chest Computed Tomography (CT) or ultrasound) or clinical/laboratory data; (2) studies that validate or evaluate methodologies and classification models for the diagnosis or outcome of patients with COVID-19. In addition, as inclusion criteria, studies published from 2019 to October 2020, in English, written in scientific format and available in full format, were established.

The following exclusion criteria were used: (1) studies related to diseases other than COVID-19; (2) studies that consider other types of information that are not images and clinical data, such as meteorological and regional data; (3) studies with a different focus of diagnosis and outcome and; (4) reviews, letters, conference abstracts, book chapters or studies that described only the application of the classification model, without its evaluation.

In addition, significant criteria regarding the application of classification methodologies and performance analysis were considered as exclusion criteria: (5) studies that did not satisfactorily describe the adopted AI method or data treatments; (6) studies that did not present sufficient description regarding the database and; (7) studies that describe results considering an unbalanced database.

### Databases and Search Strategy

The works were identified through an individual search strategy for each of the following electronic databases: ACM Digital Library, IEEE Xplore, PubMed, SCOPUS and Springer. Reference lists of selected studies were manually analyzed to search for potentially relevant studies that could have been missed in electronic searches in the databases.

For the selection and analysis, the collection of information was carried out according to keywords and their synonyms, which were used to elaborate a search string: (i) artificial intelligence; (ii) machine learning; (iii) deep learning; (iv) COVID-19; (v) 2019-nCoV; (vi) coronavirus; (vii) coronavirus infections and; (viii) new coronavirus. The selection of the most relevant articles, in accordance with the objectives of this work, from the selected keywords, Boolean operators were used to formulate the search expression, as shown in Table 1. It is worth mentioning that the search string was used for the Title/Summary fields to specify and limit the studies found for the analysis.

**Table 1:** Search string applied to selected databases

Search string	
(“artificial intelligence” **OR** “machine learning” **OR** “deep learning”) **AND** (“COVID-19” **OR** “2019-nCoV” **OR** “Coronavirus” **OR** “Coronavirus Infections” **OR** “New Coronavirus”)	

Duplicate references were removed using Mendeley^®^ software.^2^ All searches in the electronic databases occurred on October 23, 2020. The collection process searched for documents from the beginning of the pandemic.

### Selection Steps

The selection of studies was carried out in two phases. In Phase 1, all studies identified in the electronic databases were divided among 6 researchers that analyzed the titles and abstracts independently and selected those that seemed to meet the inclusion criteria. In Phase 2, these selected studies were divided among the same researchers proceeding with the complete reading independently and excluding those who did not meet the inclusion criteria. Any disagreements in the first or second phase were resolved by discussion and consensus with all researchers. In some cases, it was necessary to involve a third researcher evaluating the article to make a final decision.

**Fig. 1:**
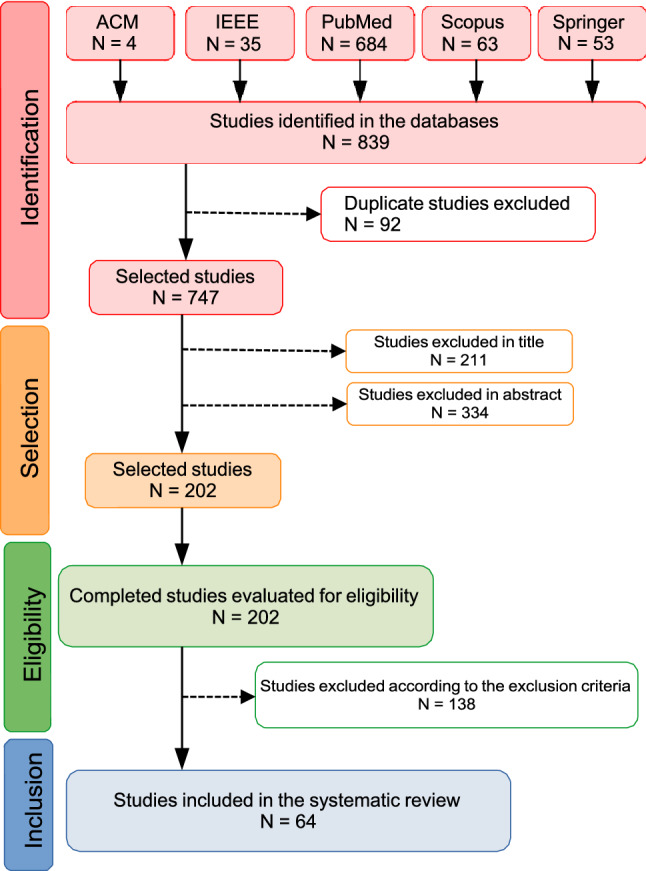
Flowchart of the study search and selection process (adapted from preferred reporting items for systematic reviews and meta-analyses—PRISMA) [20])

The first search collected 839 papers. Next, the abstracts were reviewed, and 138 remained after performing the inclusion/exclusion criteria. After thorough reading and review by a second group of reviewers, 64 met the study objectives. Figure 1 presents a flowchart with the steps and results of the PRISMA application [20].

### Study Characteristics

This section will briefly evaluate the papers that were assessed. A summary of the studies included in this systematic literature review is presented in Table 2. The Ref. column indicates the bibliographic reference, the data type column describes the type of data used in the research, the AI method column highlights the base methods used and the performance column points out the results achieved [considering the adopted metric: accuracy (A), area under the ROC curve (AUC), precision (P), sensibility(S)]. The third column indicates whether the work context was focused on diagnosis (D) or outcome (O), and finally the last column indicates the databases used in the references.

**Table 2:** Summary of an analysis of the selected studies

Ref.	Data type	AI method	Perform	D/O	Data collect from
[21]	Image (X-ray)	CNN and LSTM	99.40 (A)	D	COVID-19 image data collection. http://arxiv.org/abs/2003.11597 COVID-19 chest X-ray. https://github.com/agchung Covid-19. https://radiopaedia.org/ Cancer imaging archive (TCIA). https://www.cancerimagingarchive.net/ COVID-19 database | SIRM. https://www.sirm.org/en/category/articles/covid-19-database/ Mendeley data—augmented COVID-19 X-ray images datasethttps://data.mendeley.com/datasets/2fxz4px6d8/4 https://www.kaggle.com/paultimothymooney/chest-xray-pneumonia https://www.kaggle.com/nih-chest-xrays/data?select=Data_Entry_2017.csv
[22]	Image (CT)	CNN	91.34 (A)	D	https://github.com/ieee8023/COVID-chestxray-dataset https://www.kaggle.com/tawsifurrahman/covid19-radiography-database https://nihcc.app.box.com/v/ChestXray-NIHCC
[23]	Image (X-ray)	CNN	98.97 (A)	D	The COVID-19 Radiology database was generated: the Italian Society of Medical and Interventional Radiology (SIRM) COVID-19 Database: https://www.sirm.org/category/senza-categoria/covid-19/ Novel Corona Virus 2019 Dataset: https://github.com/ieee8023/covid-chestxray-dataset COVID-19 positive chest X-ray images from different articles:https://data.mendeley.com/datasets/rscbjbr9sj/2?__hstc=25856994.c30284243b41c8c3228c0fcd641cb3e6.1676942543457.1676942543457.1676942543457.1&__hssc=25856994.1.1676942543457&__hsfp=2139439434
[24]	Image (CT/X-ray)	CNN and ConvLSTM	88.09 (AUC)	D	https://github.com/ieee8023/covid-chestxray-dataset https://github.com/UCSDAI4H/ COVID-CT
[25]	Image (X-ray)	CNN	98.3 (P)	D	https://github.com/ieee8023/covid-chestxray-dataset
[26]	Image (X-ray)	CNN,VGG DenseNet Resnet,	99.9 (A)	D	https://github.com/ieee8023/covid-chestxray-dataset
[27]	Image (X-ray)	CNN	89 (A)	D	https://github.com/lindawangg/ COVID-Net
[28]	Image (X-ray)	CNN, Resnet, VGG	94.3 (A)	D	https://www.kaggle.com/paultimothymooney/chest-xray-pneumoniahttps://www.kaggle.com/datasets/andrewmvd/ convid19-x-rays https://data.mendeley.com/datasets/rscbjbr9sj/2
[29]	Clinical labor image (CT)	Support Vector Machine	80.95 (S)	O	https://github.com/ieee8023/covid-chestxray-datasethttps://radiopaedia.org/search?utf8=%E2%9C%93&q=covid&scope=all&lang=ushttp://www.sirm.org/en/
[30]	Clinical	Multi-Layer Perceptron	86.25 (A)	O	West London teaching hospital admissions for SARS-CoV-2 (dataset not available)
[31]	Clinical/labor	Multivariate logistic regression	95.21 (AUC)	O	Data extracted from electronic medical records of inpatients with COVID-19 in Union Hospital, Wuhan—China (dataset not available)
[32]	Image (CT)	EfficientNet B4	96 (A)	D	Data collected from 9 Chinese and 2 American hospitals (dataset not available)
[33]	Image (CT)	ResNet-50	99.87 (A)	D	(Not available): Wonkwang University Hospital Chonnam National University Hospital and Italian Society of Medical and Interventional Radiology public database
[34]	Image (CT/X-ray)	Inception V3	79.8 (S)	D	COVID-19 positive X-Rays were obtained from: https://github.com/ieee8023/ covid-chest Xray-dataset: consisting of images from a variety of sources such as case notes and publications Italian Society of Radiology [https://sirm.org/en/category/articles/%20covid-19-database/ Shenzhen Hospital (Guangdong Medical College, Shenzhen, China) http://archive.nlm.nih.gov/repos/chestImages.php
[35]	Image (CT)	U-Net	53 (A)	D	https://nihcc.app.box.com/v/ChestXray-NIHCC]—COVID-CT-Dataset [https://github.com/UCSD-AI4H/COVID-CT] Italian Society of Radiology https://sirm.org/en/ category/articles/ %20covid-19-database/
[36]	Image (ultrasound)	Deeplabv3 +	97 (A)	D	(Not available): the Italian COVID-19 Lung Ultrasound DataBase (ICLUS-DB) were acquired within different clinical centers (BresciaMed, Brescia, Italy, Valle del Serchio General Hospital, Lucca, Italy, Fondazione Policlinico Universitario A. Gemelli IRCCS, Rome, Italy, Fondazione Policlinico Universitario San Matteo IRCCS, Pavia, Italy, Tione General Hospital, Tione (TN), Italy)
[37]	Image (X-ray)	MH-CovidNet	99.38 (A)	D	https://github.com/ieee8023/covid-chestxray-dataset https://doi.org/10.17632/rscbjbr9sj.2 https://www.kaggle.com/paultimothymooney/chest-xray-pneumonia?
[38]	Clinical/Laboratory	Logistic regression	97 (AUC)	O	Across 9 clinics and hospitals within the FHC network at NYU Langone (nota available) Zhongnan Hospital of Wuhan University (nota available) https://static-content.springer.com/esm/art%3A10.1038%2Fs42256-020-0180-7/MediaObjects/42256_2020_180_MOESM3_ESM.zip
[39]	Image (CT)	uAI	N/A	O/D	Data collected from (nota available): Huoshenshan hospitals in Hubei Providence, China
[40]	Image (CT)	VGG-16	87.50 (P)	D	https://github.com/ieee8023/COVID-chestxray-dataset https://nihcc.app.box.com/v/ChestXray-NIHCC
[41]	Image (CT)	EfficientNet	87.50 (AUC)	D	https://data.mendeley.com/datasets/3y55vgckg6/1
[42]	Image (CT)	kNN	87.75 (A)	D	http://medicalsegmentation.com/covid19/ https://radiopaedia.org/articles/covid-19-3 https://radiopaedia.org/cases/covid-19-pneumonia-3, https://radiopaedia.org/cases/covid-19-pneumonia-8, https://radiopaedia.org/cases/covid-19-pneumonia-23 https://radiopaedia.org/cases/covid-19-pneumonia-10, https://radiopaedia.org/cases/covid-19-pneumonia-27, https://radiopaedia.org/cases/covid-19-pneumonia-52 https://radiopaedia.org/cases/covid-19-pneumonia-55, https://radiopaedia.org/cases/covid-19-pneumonia-56 https://www.cancerimagingarchive.net/access-data/ http://ncia.nci.nih.gov/ https://wiki.nci.nih.gov/display/CIP/LIDC, https://doi.org/10.7937/K9/TCIA.2015.U1X8A5NR
[43]	Image (CT)	DeepLabv3	91 (AUC)	O	http://ncov-ai.big.ac.cn/download?lang=em
[44]	Image (CT)	AFS-DF	91.79 (A)	D	(nota available) by The Third Hospital of Jilin University, Ruijin Hospital of Shanghai Jiao Tong University, Tongji Hospital of Huazhong University of Science and Technology, Shanghai Public Health Clinical Center of Fudan University, Hangzhou First People’s Hospital of Zhejiang University, and Sichuan University West China Hospital
[45]	Clinical	Support Vector Machine	92 (A)	D	https://github.com/BDBC-KG-NLP/COVID-19-tracker
[46]	Image (CT)	Ensemble	91.94 (A)	D	(not available):—Iran University of Medical Sciences (IUMS), Tehran, Iran Faculty of Health and Medical Sciences,Taylor’s University, 47500 Subang Jaya, Malaysia Faculty of Medicine, Islamic Azad University, Tehran Medical Sciences Branch, Tehran, Iran Faculty of Medicine, Urmia University of Medical Science, Urmia, Iran
[47]	Image (X-ray)	Capsule network	97.24 (A)	D	https://nihcc.app.box.com/v/ChestXray-NIHCC https://github.com/ieee8023/COVID-chestxray-dataset
[48]	Image (CT)	Logistic regression	97 (AUC)	O	(not available) from hospitals in Ankang, Lishui, Lanzhou, Linxia,and Zhenjiang (all in China)
[49]	Clinical/labor	Logistic regression	89.50 (A)	O	Hospital Sino-French New City Branch of Tongji Hospital, Wuhan, China
[50]	Image (CT)	COVNet	96 (S)	D	Wuhan Huangpi People's Hospital, Wuhan, Hubei, China 430301—Huangpi People's Hospital, Wuhan, Hubei, China 430301—Wuhan Pulmonary Hospital, Wuhan, Hubei, China 430,030 -Liaocheng People's Hospital, Shandong, China, 252000—The Third Medical Center of Chinese PLA General Hospital, Beijing, China 100039—Shenzhen Second People's Hospital/the First Affiliated Hospital of Shenzhen University Health Science Center, Shenzhen, China 518035
[51]	Labora-tory	ANN, CNN, LSTM, RNN, CNNLSTM and CNNRNN	86.66 (A)	D	https://www.kaggle.com/datasets/einsteindata4u/covid19
[52]	Clinical/labor./image (CT)	Random forest, Logist regression and U- Net	89.90 (A)	O	Data collected from (not available): Data collected from First Affiliated Hospital at Zhejiang University School of Medicine
[53]	Image (CT)	ResNet-101	99.63 (A)	D	Hospital, Iran University of Medical Sciences (IUMS), Tehran, Iran Razi Hospital, Guilan University of Medical Sciences, Rasht, Iran Hospital, Faculty of Health and Medical Sciences, Taylor's University, 475(X), Subang Jaya, Malaysia Hospital, Faculty of Medicine, Urmia University of Medical Science, Urmia, Iran
[54]	Image (CT)	ResNet-50	95.18 (P)	D	Various datasets such as from [https://www.sirm.org/category/senza-categoria/covid-19/] [https://github.com/ieee8023/covid-chestxray-dataset] and [6 hospitals in Anhui province, China],
[55]	Image (X-Ray)	VGG-19	89.3 (A)	D	https://github.com/ieee8023/covid-chestxray-dataset https://www.kaggle.com/datasets/paultimothymooney/chest-xray-pneumonia
[56]	Image (CT)	U-Net	91.03 (A)	D	http://medicalsegmentation.com/covid19/ Henri Becquerel Cancer Center (HBCC) in Rouen, France—https://github.com/UCSD-AI4H/COVID-CT
[57]	Image (X-Ray)	VGG-16	88.10 (A)	D	https://radiopaedia.org/search?utf8=%E2%9C%93&q=covid&scope=all&lang=us http://www.sirm.org/en/
[58]	Image (X-Ray)	DenseNet-121	88 (S)	D	(Not avaliable)
[59]	Image (CT)	U-Net	*≈* 97 (A)	D	https://github.com/JoHof/lungmask
[60]	Image (CT)	Inf-Net	87 (P)	D	https://medicalsegmentation.com/covid19/ https://www.kaggle.com/nabeelsajid917/covid-19-x-ray-10000-images https://github.com/ieee8023/covid-chestxray-dataset -https://github.com/UCSD-AI4H/COVID-CT https://www.kaggle.com/datasets/tawsifurrahman/covid19-radiography-database
[61]	Clinical/Labor	Random forest	83.4 (A)	O	(Not avaliable)
[62]	Image (CT)	ResNet-50	95.21 (A)	D	(Not available): Shanghai Public Health Clinical Center, Fudan University
[63]	Image (CT)	U-Net	89.2 (A)	D	Hospital of Wuhan Red Cross Society (WHRCH) Shenzhen Second Hospital (SZSH) https://wiki.cancerimagingarchive.net/pages/viewpage.action?pageId=24284539
[64]	Image (CT)	AD3D0MIL	97.9 (P)	D	(Not available) from hospitals in Shandong Province, China
[65]	Image (CT)	ResNet	86.70 (A)	D	First Affiliated Hospital, College of Medicine, Zhejiang University, Zhejiang Province, China Wenzhou Central Hospital, Zhejiang Province, China First People’s Hospital of Wenling, Zhejiang Province, China
[66]	Image (X-Ray)	ResNet50/Resnet101	97.77 (A)	D	https://github.com/ieee8023/covid-chestxray-dataset (not available):—Henry Ford Health System https://www.kaggle.com/datasets/paultimothymooney/chest-xray-pneumonia
[67]	Image (X-Ray)	EfficientNets	56.16 (A)	D	https://www.kaggle.com/plameneduardo/sarscov2-ctscan-dataset https://github.com/UCSD-AI4H/COVID-CT
[68]	Clinical/Laboratory	Logistic regression	96.2	D	https://github.com/Akibkhanday/Meta-data-of-Coronavirus
[69]	Image (CT)	DenseNet201	96.25 (A)	D	www.kaggle.com/plameneduardo/sarscov2-ctscan-dataset
[70]	Image (X-Ray)	VGG16	95.88 (A)	D	https://github.com/ieee8023/covid-chestxray-dataset https://www.kaggle.com/paultimothymooney/chest-xray-pneumonia https://github.com/AntonisMakris/COVID19-XRay-Dataset
[71]	Image (X-Ray)	EfficientNet-B0	99.62 (A)	D	https://www.sirm.org/category/senza-categoria/covid-19/-https://github.com/ieee8023/covid-chestxray-dataset -https://www.kaggle.com/tawsifurrahman/ covid19-radiography-database https://radiopaedia.org/search?lang=us&page=4&q=covid+19&scope-all&utf8=%E2%9C%93 https://threadreaderapp.com/thread/ 1243928581983670272.html https://nihcc.app.box.com/v/ChestXray-NIHCC https://www.kaggle.com/paultimothymooney/chest-xray-pneumonia
[72]	Image (X-ray)	VGG16	96 (S)	D	https://github.com/ieee8023/covid-chestxray-dataset https://github.com/muhammedtalo/COVID-19 https://www.kaggle.com/nih-chest-xrays/sample
[73]	Image (CT)	Ensemble	86 (A)	D	https://github.com/UCSD-AI4H/COVID-CT
[74]	Image (CT)	U-Net	78.3 dice	D	4 different hospitals in Heilongjiang Province, China King Faisal Specialist Hospital and Research Center (KFSHRC) in Riyadh, Saudi Arabia
[75]	Image (CT)	SqueezeNet	85.03 (A)	D	https://sirm.org/category/senza-categoria/covid-19/ https://github.com/UCSD-AI4H/COVID-CT
[76]	Image (X-ray)	ResNet-18	88.9 (A)	D	www.macnet.or.jp/jsrt2/cdrom_nodules.html https://www.kaggle.com/praveengovi/coronahack-chest-xraydataset, https://github.com/ieee8023/covid-chestxray-dataset
[77]	Image (X-ray)	Inception V3	100, 85, 76 (A)	D	https://github.com/ieee8023/covid-chestxray-dataset -QUIBIM imagingcovid19 platform database and various public repositories, including RSNA, IEEE, RadioGyan and the British Society of Thoracic Imaging
[78]	Image (CT)	ResNet50	84.9 (A)	D	https://github.com/UCSD-AI4H/COVID-CT
[79]	Image (X-ray)	Grad-CAM	90.02 (A)	D	Guangzhou Medical Center, China Sylhet Medical College, Bangladesh
[80]	Laboratory	Logistic regression	98 (S), 91 (P)	O	(Not available): Tongji Hospital of Tongji Medical College of Huazhong University of Science and Technology, Wuhan, China
[81]	Image (CT)	DenseNet-201	95.2 (A)	O	First Affiliated Hospital of Bengbu Medical College First Affiliated Hospital of Anhui Medical University Fuyang Second People’s Hospital
[82]	Image (X-ray)	Decision tree	98 (A)	D	https://nihcc.app.box.com/v/ChestXray-NIHCC/folder/36938765345 https://lhncbc.nlm.nih.gov/publication/pub9931 https://nihcc.app.box.com/v/ChestXray-NIHCC/folder/36938765345 Eastern Asia Hospital dataset
[83]	Clinical/laboratory	XGboost	84 (A)	O	Vulcan Hill Hospital, located in Wuhan, Hubei Province, China (not available)
[84]	Image (CT)	Ensemble	94.16 (A)	D	Ruian People’s Hospital, China (not available)

*A* accuracy, *AUC* area under the roc curve, *P* precision, *S* sensibility, *D* diagnosis, *O* outcome

Some works pointed out in Table 2 as AI Method, used a generic nomenclature of deep learning (DL) algorithms, such as recurrent neural networks (RNN) and convolution neural networks (CNN) as indicated in references [21-28]. CNNs is the generic name of the DL-based algorithm that introduced the convolution process in the neural network area [85], inspired by the animal visual cortex, which is most applied to in image analysis. CNNs learn to extract features from data while learning to correlate these features with the labels to minimize the losses between model and label output.

On the other hand, RNNs were created as a class of artificial neural networks that allow connections between neurons of the same layer or between non-adjacent layers generating links that form oriented or non-oriented graphs over a temporal sequence. More recently, model-based DL architectures have been inserted into this class of algorithms that aim to extract information from data that are related to longer temporal chains of information [86].

Other works used models based on hybrid architectures, combining more than one architecture of deep neural networks, such as CNNRNN (combination of CNN and RNN) or CNNLSTM (combination of CNN and LSTM (long-short time memory) indicated in reference [51]. The papers [26, 28] are essential to highlight that used eleven and five, respectively, different types of CNN-architectures such as VGG (classical CNN), residual neural network (Resnet), Inception, etc., to build a framework involving these models. As can be seen in Table 2, most of the papers aim at diagnosis because the first test for COVID-19 diagnosis was released in February 2020, distributed by the Centers for Disease Control and Prevention. However, many countries with limited resources prevented testing all patients with suspected COVID-19, which stimulated the search for alternative methods for diagnosis, either with imaging or laboratory tests.^3^ Thus, only about 20% of the studies involved the evaluation of the outcome.

**Fig. 2:**
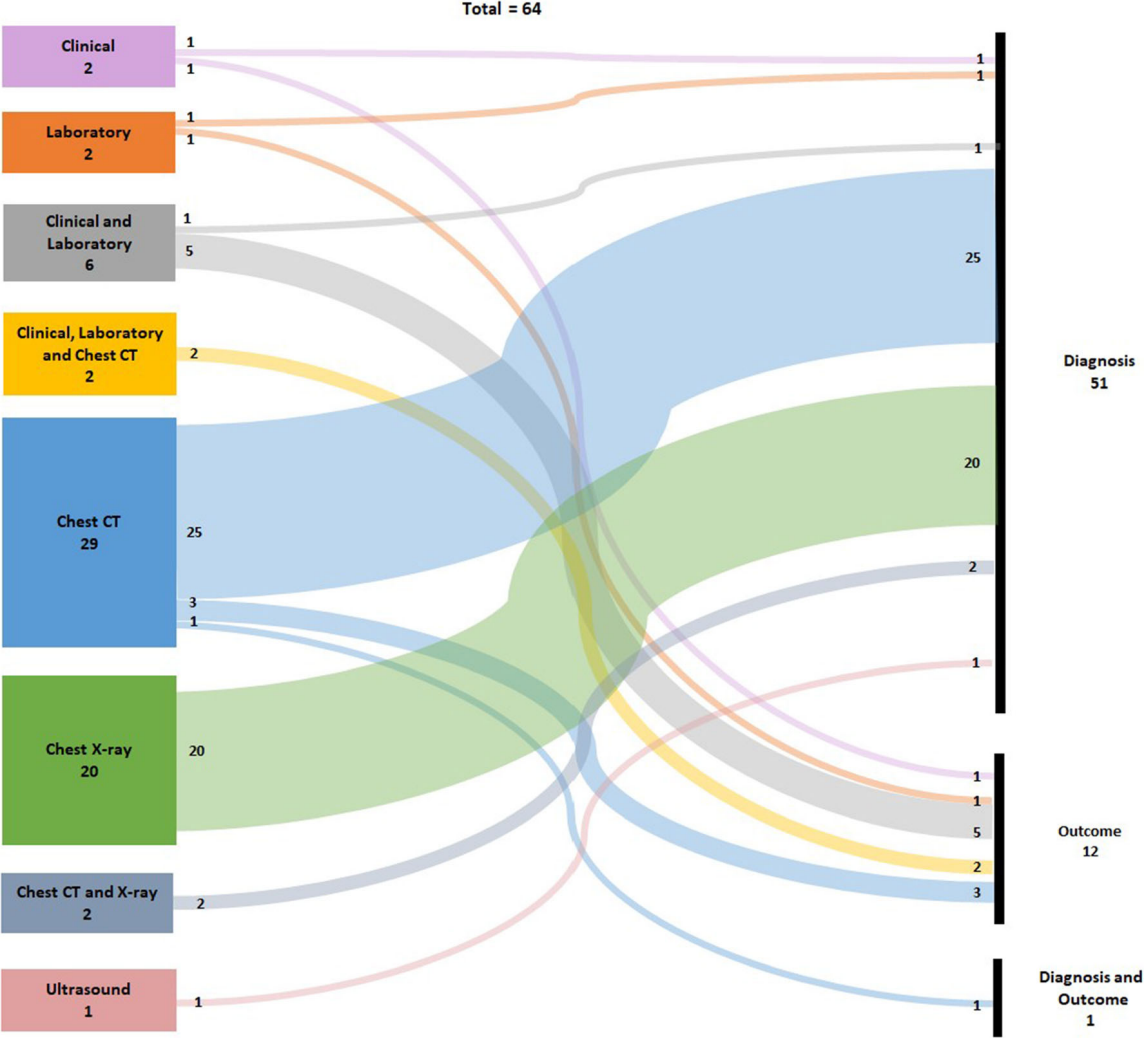
Distribution of papers concerning their data types and purpose

The Sankey diagram represented in Fig. 2 presents the frequency distribution of the papers concerning the data types (clinical, laboratory, chest X-ray, chest CT, ultrasound) and their purpose (diagnosis, outcome and diagnosis/outcome). The majority of the selected papers reached the diagnosis purpose (51 papers) while 12 worked on the Outcome problem and only one considered both situations.

The most used data type was chest CT scans, 29 papers, followed by chest X-Ray in 20 works and then a combination of Clinical and Laboratory data in six articles. Two studies applied Clinical or Laboratory data. The combination of Clinical, Laboratory and chest CT scans data are found in two works, and the combination of chest CT and chest X-Ray data are also found in another two works, respectively. Only one work used Ultrasound data.

Another point to be highlighted in the Fig. 2 is that while most of the data types were used to approach one or two kinds of purpose (diagnosis, outcome or both), only the chest CT scan was used on all of them. Fig. 2, when obtaining the relationship between the types of data and purposes, two thicker lines are identified, the data from CT and X-ray images, which led to the diagnosis (70.31%). Although CT is more accurate, X-ray is widely used due to its avail- ability and ease of access, especially in bedridden patients. In the beginning of COVID-19, the disease was considered to be strongly related to lung problems.

**Fig. 3:**
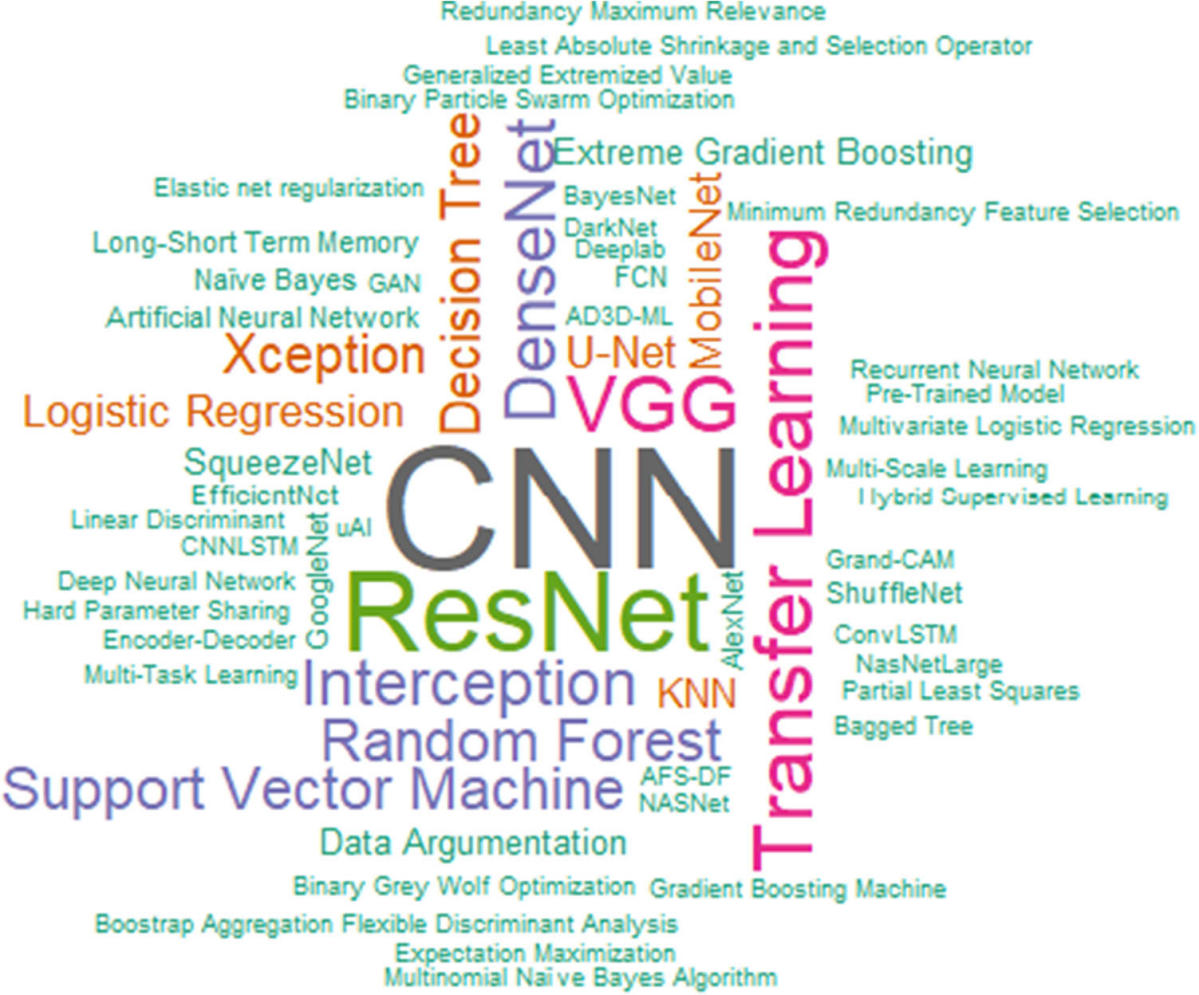
Word cloud generated by AI terms that appeared in the selected studies

Figure 3 presents a word cloud generated with several AI-based techniques and algorithms used in the analyzed selected studies.

Words written in larger size indicate their expressiveness in relation to their distribution of frequency in the selected studies. Thus, we can conclude that most of the works involve image examinations because CNN [21-28, 51] and Resnet [26, 28, 33, 53, 54, 62, 65, 66, 76, 78] (which are associated with CNN) are the largest size words. In following, the largest words are transfer learning [55, 62, 66, 69, 70, 76, 77] and VGG [26, 28, 40, 55, 57, 70, 72]. There is an emphasis on the use of Transfer Learning because many researchers have had to reduce the time to produce results for COVID-19 diagnosis (reducing training time and the amount of labeled data needed for training) and VGG was another highlighted technique mainly for being a CNN used in image classification.

## Discussions

### Images Papers

The Fig. 2 shows all selected articles divided in groups by their data where 52 are based only on different types of chest images. Among all, 20 studies examined chest X-Ray scans [21, 23, 25-27, 29, 37, 47, 55, 57, 58, 66, 67, 70-72, 76, 77, 79, 82], 29 studies focused in chest CT [22, 32, 33, 35, 39-44, 46, 48, 50, 53, 54, 56, 59, 60, 62-65, 69, 73-75, 78, 81, 84], two studies considered both chest X-ray and chest CT [24, 34] and one study focused in ultrasound [36].

**Fig. 4:**
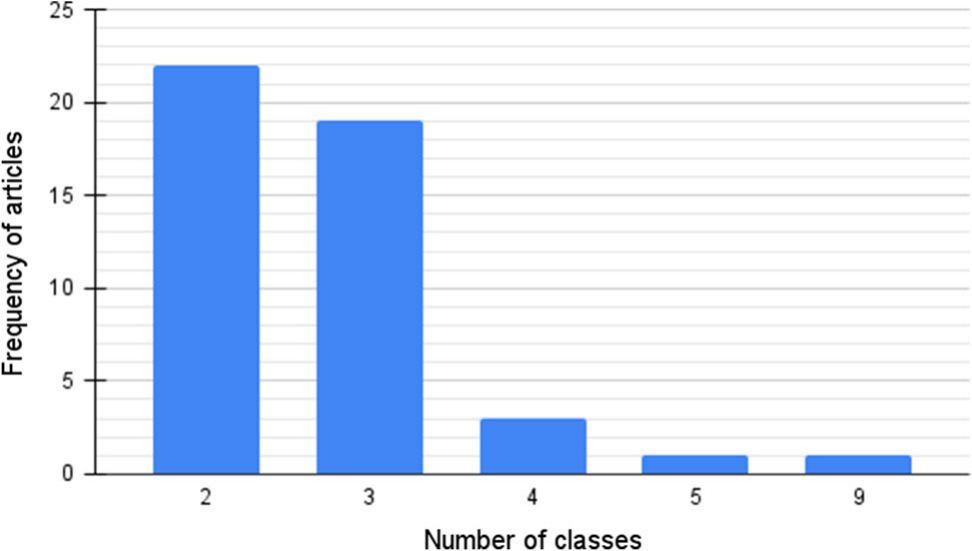
Frequency of articles versus number of output classes evaluated

In order to compare the different data preprocessing techniques, present in the articles, we named three main characteristics: resize is related to the scale (in px) of the input images; synthetic means the use of GAN’s to create synthetic data and; Increase indicates the image number increase by data-augmentation techniques, which consists of making small changes to the original images, such as color and brightness changes, shift, flip and image rotations, as well as cropping. Table 3 relates the articles to their respective data preprocessing characteristics. Among the 52 selected articles, 45 perform classification tasks with the number of output classes ranging from two to nine. Most of the articles adopted two or three classes, as one can see in Fig. 4, which shows the histogram of the distribution of frequency of articles versus number of output classes. COVID-19 is the only class shared by the 45 articles, Pneumonia and Healthy are the most common Non-COVID-19 classes among them.

**Table 3:** Approaches related to data preprocessing

References	Resize	Synthetic	Increase
[24, 27, 57]	Yes	Yes	Yes
[71]	Yes	Yes	No
[21, 26, 32, 33, 47, 55, 60, 62-66, 69, 70, 72, 74-78, 82, 84]	No	Yes	Yes
[28, 35, 42, 43, 53, 56, 58, 67, 73, 79, 81]	Yes	No	No
[23]	No	No	Yes
[22, 25, 34, 36, 37, 39-41, 44, 46, 48, 50, 54, 59]	No	No	No

However, some authors consider other illnesses, like bacterial pneumonia [26], H1N1 [62], influenza [65] and tuberculosis [82], as well as some authors consider illness stages, as in [27] and [66]. [64] evaluate the proposed model by distinguishing two classes (COVID-19 and non-COVID-19) and then three classes (COVID-19, common pneumonia, and no pneumonia). So, this article is considered in the two respective columns in Fig. 4.

Tables 4 and 5 present the number of COVID-19 and Non-COVID-19 images for each of the 45 articles. These numbers are the articles’ original data quantities before data preprocessing, which means that the data imbalance present in some rows of Tables 4 and 5 do not represent the number of images used to train the proposed models. For instance, [81] does not employ non-COVID- 19 images, as shown in Table 5, because the authors use chest CT images for the assessment of the illness severity in COVID-19 infected patients. The non-COVID-19 images refer to healthy, pneumonia or any other illness or class considered by the different authors. It is important to consider that [34] and [24] employ both chest X-ray and CT images on their work. So, they are mentioned in both Tables 4 and 5.

**Table 4:** Number of chest X-ray images used, by class, in the databases evaluated in the papers

References	COVID	Non-COVID	(%)
[58]	5805	5300	52.27
[28]	524	504	50.97
[79]	305	305	50.00
[83]	240	240	50.00
[57]	158	158	50.00
[25]	30	30	50.00
[24]	28	28	50.00
[67]	1601	1693	48.60
[21]	1525	3050	33.33
[37]	364	728	33.33
[22]	500	1000	33.33
[40]	100	200	33.33
[70]	112	224	33.33
[55]	260	600	30.23
[26]	69	237	22.55
[77]	122	450	21.33
[66]	440	1862	19.11
[47]	231	2100	9.91
[71]	263	3223	7.54
[30]	219	2686	7.54
[72]	250	6003	4.00
[76]	360	15,185	2.32
[27]	337	35,319	0.95
[34]	129	62,267	0*.*21

**Table 5:** Number of Chest CT images used, by class, in the databases evaluated in the papers

References	COVID	Non-COVID	(%)
[81]	729	0	100.00
[39]	2376	84	96.59
[84]	615	146	80.81
[44]	1495	1027	59.28
[75]	888	783	53.14
[69]	1262	1230	50.64
[24]	25	25	50.00
[42]	200	200	50.00
[46]	306	306	50.00
[53]	510	510	50.00
[64]	230	230	50.00
[41]	23,579	23,849	49.72
[32]	65,807	66,777	49.63
[54]	413	439	48.47
[73]	360	397	47.56
[78]	349	393	47.04
[43]	164,241	279,793	36.99
[65]	219	399	35.44
[56]	449	920	32.80
[33]	1194	2799	29.90
[50]	1296	3060	29.75
[62]	32,301	86,435	27.20
[34]	229	2088	9.88

Through the 52 selected articles, five achieve segmentation tasks, one performs predictions of hospitalization stay and one predicts the disease severity score. Reference [63] presents a net architecture that executes lung segmentation and illness classification. The segmentation dataset consists of 60 3D CT lung scans with manual delineations of the lung anatomy. The classification dataset included 150 3D volumetric chest CT exams of COVID-19, Community Acquired Pneumonia and non-pneumonia patients. However, the authors do not specify each class ‘selected slice number for training/validation and testing.

In order to propose COVID-19 segmentation models, [35, 74] employ datasets that consist of only infected patients chest CT scans, but they do not specify the number of images per CT scan. Reference [60] include non-infected CT scans to guarantee that the proposed model deals with non-infected slices well. Reference [59] employ COVID- 19 infected patients and patients with different medical histories other than COVID-19 to perform lung segmentation and quantification of lung opacities in chest CT scans of COVID-19 patients.

Yu et al. [81] include 31 patients with 72 lesion segments for predicting hospital stay in infected patients using chest CT scans. But it is not clear if the number of lesion segments is the number of images or slices.

An approach that uses DL techniques for the analysis of lung ultrasonography images is proposed by [36]. The authors propose a model that employs 277 lung ultrasound videos from 35 patients, corresponding to 58,924 frames. This model is a deep network that simultaneously predicts the disease severity score associated to a input frame and provides localization of pathological artefacts in a weakly-supervised way.

A relevant point about works [53, 67, 84], despite having included images as part of the data used in the research, unlike the other works, they did not use CNN to extract information from the images. Instead, the authors of these works used specific filters to extract the spatial distribution of signal intensities and pixel interrelationships [87]. The radiomics, which quantifies textural in formation, is a method that extracts, in general, a large number of features from medical images using data-characterization algorithms. [84] also used radiomics method to collect 34 statistical texture features of COVID- 19 and general pneumonia ROI images, aiming to differentiate COVID-19. The authors used ensemble of bagged trees and compared their results with other ML algorithms (K-nearest neighbor (kNN), decision tree, support vector machine (SVM), logist regression (LR)).

Four different algorithms were evaluated (decision tree, kNN, Naïve Bayes, SVM) by [46] and ensemble-based algorithms involved the tree-based algorithms: AdaBoost, RUSBoost, LogitBoost, GentleBoost and bag. In this work, the features of the images were also not extracted by filters or CNNs algorithms. The radiological features were extracted by radiologists during the visual inspection process.

In the paper by [56] the lung volume, lesion volume, nonlesion lung volume (lung volume—lesion volume) and the fraction of nonlesion lung volume were quantified by U-Nets. In addition, models based on Random Forest (RF) and LR were developed to classify and assess disease severity and predict the length of ICU stay, the duration of oxygen inhalation, hospitalization, and patient prognosis using clinical data.

In turn, the paper by [73] used several CNN architectures (VGG16, InceptionV3, ResNet50, DenseNet121 and DenseNet201) keeping the original convolutional parts as in the standard models and changed the fully connected parts of the models, fixed as tree fully connected layers to evaluate the diagnosis of COVID-19 patients. The individual results of these models were merged via a majority voting approach.

### Clinical and Laboratorial Papers

Of the papers selected in this review, ten works address clinical data, and ten have laboratory data. The patients’ demographic data, symptoms and history of diseases were considered clinical data. Figure 2 shows the distribution of articles regarding two variables: (i) diagnosis or outcome and (ii) the type of data: clinical, laboratory or imaging data (chest CT, chest X-ray or ultrasound). In the clinical data, there were one paper discussing diagnosis [45] and one concerning outcome [30]. In the laboratory data, there were only one work for the diagnosis [51] and another for outcome [80]. One paper that bring together clinical and laboratory data deal with diagnosis [68] and four with outcomes [31, 38, 49, 61, 83].

**Fig. 5:**
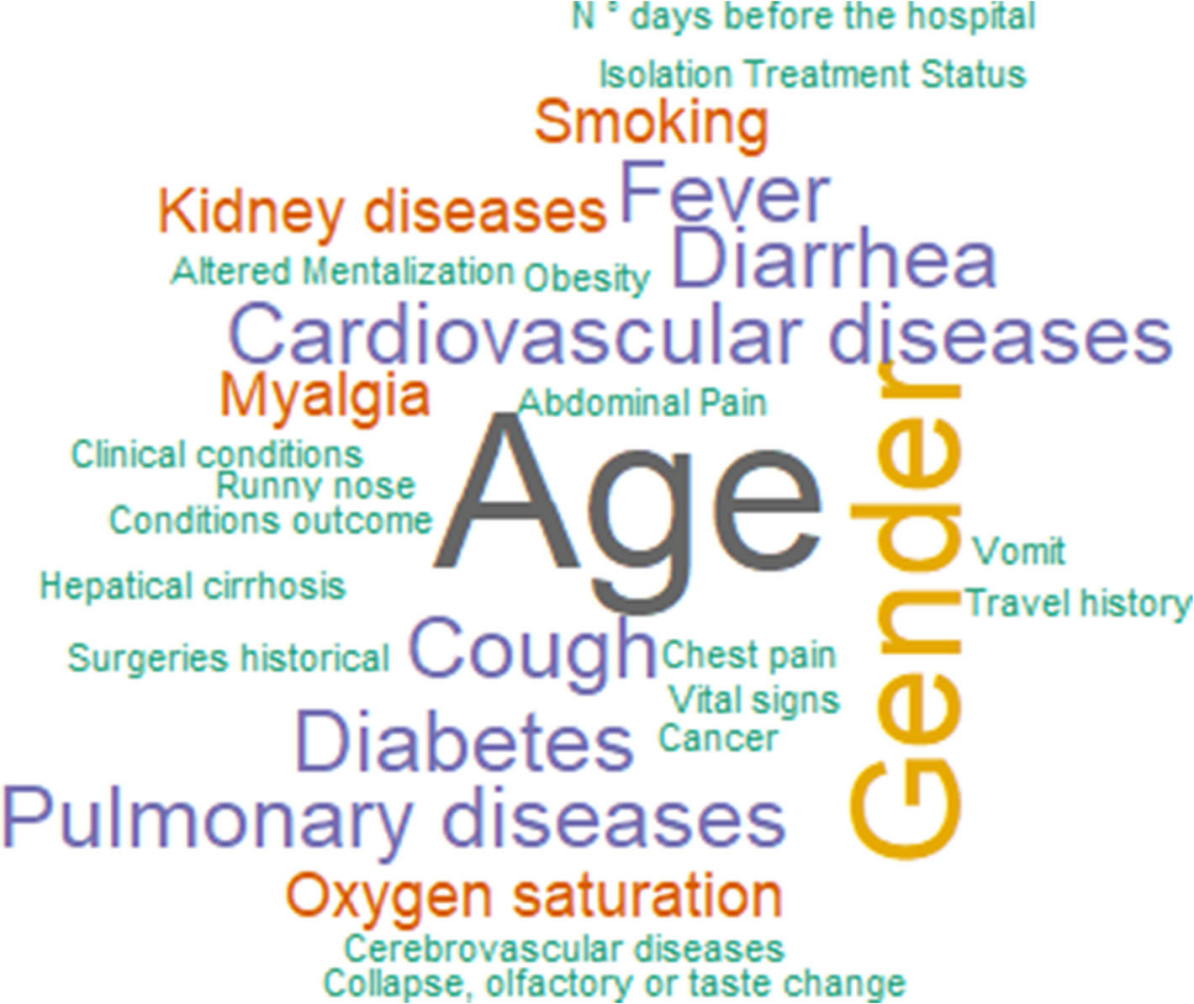
Clinical data word cloud

Figure 5 presents the clinical data word cloud. Age is included in 90% of the works [29-31, 38, 45, 49, 52, 61, 68]. Gender in 60% [31, 38, 49, 52, 61, 68]and cough [29, 38, 68], fever [31, 38, 68], diabetes [31, 49, 52], diarrhea [29, 31, 38], cardiovascular diseases [31, 49, 52] and pulmonary diseases [31, 38, 52] in 30%. Two works corresponding 20% of the clinical data papers mentioned smoking and kidney diseases [31, 52], oxygen saturation [29, 61] and myalgia symptoms [31, 38]. The other data are used only in one work.

**Fig. 6:**
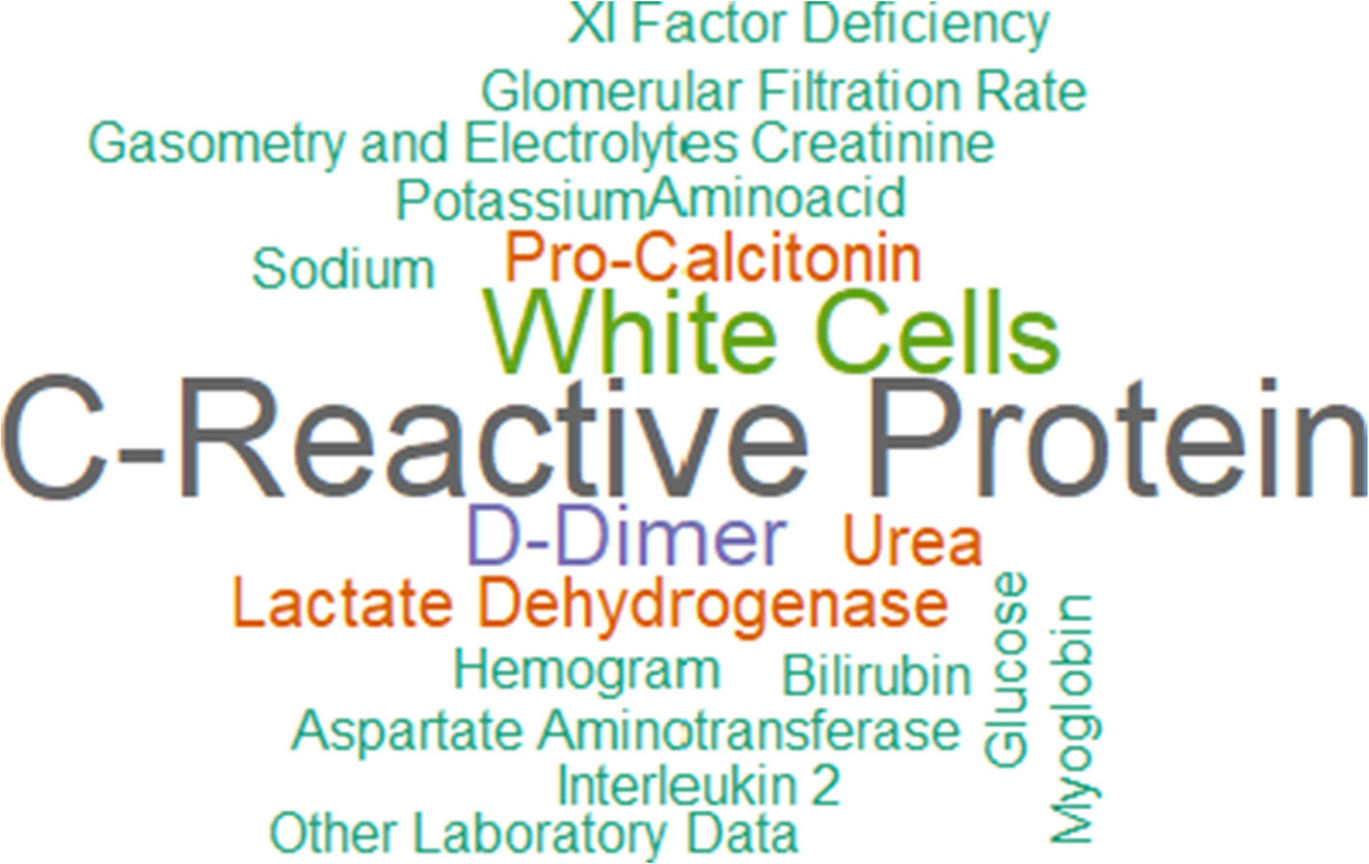
Laboratorial data word cloud

The most used laboratory tests were C-reactive protein [29, 31, 38, 49, 51, 52, 61, 83] and white blood cells [29, 49, 52, 68, 80] with means respectively 80% and 50%. Then the D-Dimer exam with three articles (30%) [38, 49, 80]. The lactate dehydrogenase [31, 83], pro-calcitonin [38, 52] and urea [44, 73] exams had 20% of the articles (two of ten). Hemogram was used in only one work [51]. Figure 6 presents the laboratory word cloud.

We observed that the works [30, 38, 45, 51, 61, 68] did not mention any kind of variable selection technique. Whereas the works [29, 52, 80, 83] adopted different strategies to identify more promising attributes among the variables that compose the collected dataset: the minimum redundancy maximum correlation algorithm [88], least absolute shrinkage and selection operator algorithm [89], Spearman’s correlation and chi-square test or Fisher exact test [90], Principal Component Analysis [91]. The papers [31, 49] used tree-based algorithms to select the most important attributes and in the case of paper [31], it adopted these selected attributes in other classification algorithms.

### Clinical, Laboratorial and Images Papers

Of the 64 selected papers, only two addressed clinical, laboratory and imaging data [29, 52]. They were related to the outcome, with data on age, pro-reactive protein, white blood series, and chest CT. [52] use U-Net and data of oxygen saturation, surgeries historical, gasometry and electrolytes, and pro-calcitonin. The different clinical data of [29] are gender, chest pain, cough, diarrhea, and laboratory data are aminotransferase and myocardial enzymes.

Since AlexNet’s publication in 2012, CNN have become ubiquitous in the world of Computer Vision [92]. Resnet [93], CNN, VGG, U-Net, and EfficientNet [94] are architectures DL and use the convolutional process to extract information from the images [95] in 56% of the papers of this study.

## Results

This Systematic review identified at first 839 papers but 24% were eligible and in the final selection there were 64 papers that represent 8% of the total papers found. The major of the papers (79.7%) has the purpose of Diagnosis, and only 12 papers (18.8%) address Outcome and only one (1.5%) both.

**Fig. 7:**
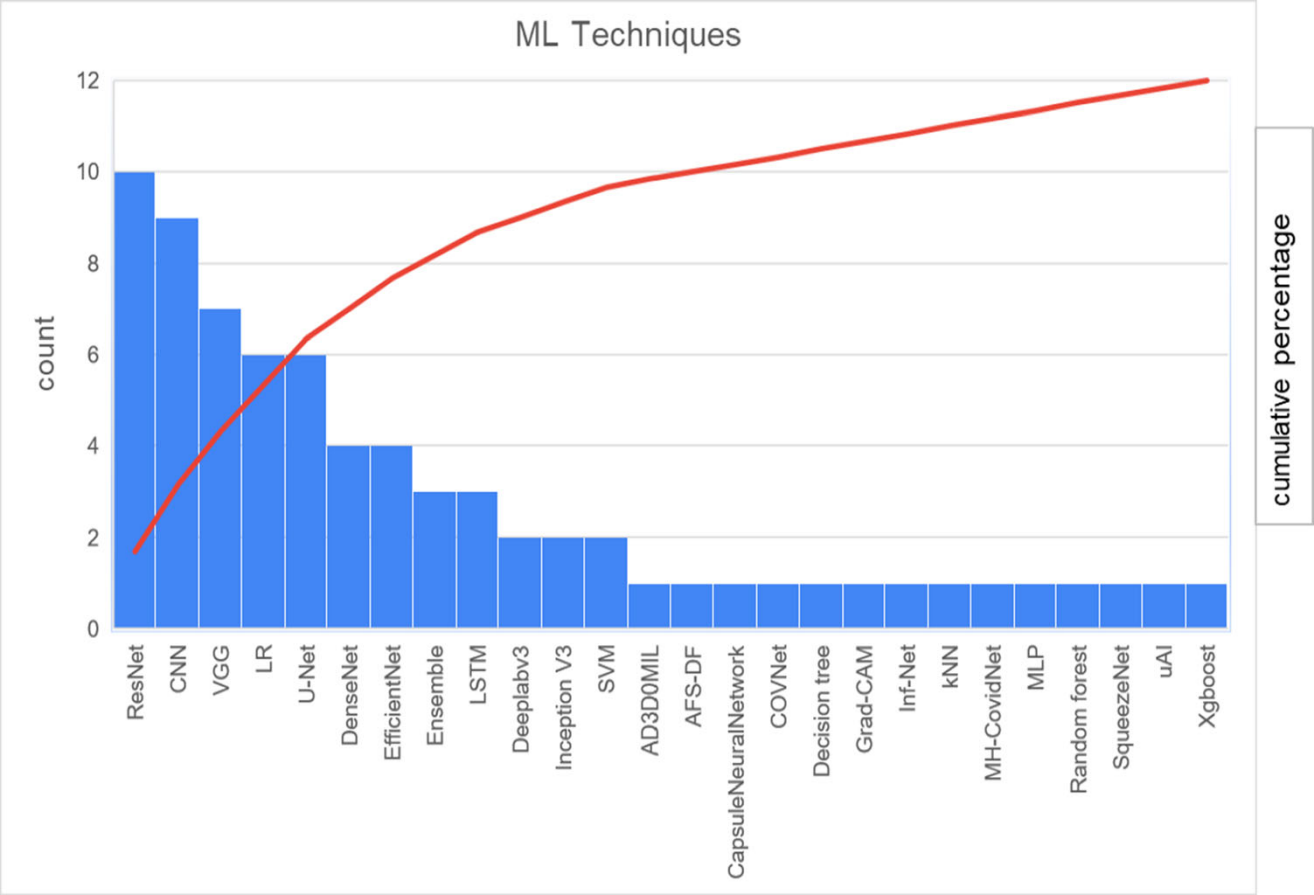
ML techniques in COVID-19

The ML techniques used by papers selected in this systematic review is summarized in Table 6. The Fig. 7 presented the machine learning techniques comparative survey, that we can highlight the following techniques: Resnet (15,6%), CNN (14,1%), VGG (10,9%), LR (9,4%), U-Net (9,4%), DenseNet (6,3%), and EfficientNet (6,3%). Since AlexNet’s publication in 2012, CNN have become ubiquitous in the world of Computer Vision [92]. Resnet, CNN, VGG, U-Net and EfficientNet are architectures DL and use the convolutional process to extract information from the images [95] in 56% of the papers of this study.

**Table 6:** Frequency of the Machine Learning techniques used in the papers

AI technique	References	Frequency
AD3D0MIL	[64]	1
AFS-DF	[44]	1
Capsule neural network	[47]	1
CNN	[21-28, 51]	9
COVNet	[50]	1
Decision tree	[82]	1
Deeplabv3	[36, 43]	2
DenseNet	[26, 58, 69, 81]	4
EfficientNet	[32, 41, 67, 71]	4
Ensemble	[46, 53, 84]	3
Grad-CAM	[79]	1
Inception V3	[34, 77]	2
Inf-Net	[60]	1
kNN	[42]	1
LR (logistic regression)	[31, 38, 48, 49, 68, 80]	6
LSTM	[21, 24, 51]	3
MH-CovidNet	[37]	1
MLP (multi-layer perceptron)	[30]	1
Random forest	[61]	1
ResNet	[26, 28, 33, 53, 54, 62, 65, 66, 76, 78]	10
SqueezeNet	[75]	1
SVM (Support Vector Machine)	[29, 45]	2
uAI	[39]	1
U-Net	[35, 52, 56, 59, 63, 74]	6
VGG	[26, 28, 40, 55, 57, 70, 72]	7
Xgboost	[83]	1

Very deep convolutional networks (VGG) is a family of architecture based on pattern recognition models. Developed as a deep neural network, it is one of the most popular image recognition architectures. The number of convolutional layers with VGG-16 or VGG-19 are 16 and 19, respectively. They are based on convolution and pooling operations along the layers, finishing with fully connected layers. The VGG-16 is composed of 13 convolutional layers, and VGG-19 has 16 convolution layers, both having three fully connected layers at the end. Specifically, the VGG architecture has increased the convolutional layers and uses a too-small filter (3x3 with a stride of 1), following the revolution caused by AlexNet with the introduction of ReLu (activation function), which won the ImageNet 2012 contest.

The “fully convolutional network” U-Net [96] have initially been developed for biomedical image segmentation at the Department of Computer Science of the University of Freiburg. The U-shape of this architecture consists of a sequence of convolutional contracting layers (the spatial information is reduced while feature information is increased) followed by another sequence in symmetric opposite with expansive character (the feature and spatial information are combined through a sequence of up-convolutions), which gives it a U-shaped architecture. The U-shape of this architecture consists of a sequence of convolutional contracting layers (the spatial information is reduced while feature information is increased). Another series of layers, which is almost symmetrical concerning the first sequence, has an expansive character (the feature and spatial information are combined through a sequence of up-convolutions), which gives it a U-shaped design. Thus, without exception, all DL networks used in image data are therefore used in work with image data, either for classification of these images for diagnosis or the outcome or semantic segmentation, which aims to identify regions affected by COVID-19 in lung images.

Only 7,8% of the papers use more than one ML techniques: paper [26] uses Resnet, CNN, VGG and DenseNet; (ii) paper [28] uses Resnet, CNN and VGG. The works [21, 24] and [51] uses CNN and LSTM.

Table 7 presents a summary of the data type and purpose (diagnosis or outcome) of the ML methods.

**Table 7:** Data type and purpose for the machine learning techniques

AI Method	Data type	D + O	References
AD3D0MIL	CT	D	[64]
AFS-DF	CT	D	[44]
Capsule network	X-ray	D	[47]
CNN	CT	D	[22]
CNN	CT + X-ray	D	[24]
CNN	Laboratory	D	[51]
CNN	X-ray	D	[21, 23, 25-28]
ConvLSTM	CT + X-ray	D	[24]
COVNet	CT	D	[50]
Decision tree	X-ray	D	[82]
DeepLabv3	CT	O	[43]
Deeplabv3+	Ultrasound	D	[36]
DenseNet	X-ray	D	[26, 58]
DenseNet	CT	D	[70]
DenseNet	CT	O	[81]
EfficientNet	CT	D	[32, 41]
EfficientNet	X-ray	D	[67, 71]
Ensemble	CT	D	[46, 73, 84]
Grad-CAM	X-ray	D	[79]
Inception V3	CT + X-ray	D	[34]
Inception V3	X-Ray	D	[77]
Inf-Net	CT	D	[60]
kNN	CT	D	[42]
Logistic regression	Clinical + laboratory	D	[68]
Logistic regression	Clinical + laboratory	O	[38, 49]
Logistic regression	CT	O	[48]
Logistic regression	Laboratory	O	[80]
Logistic regression	Clinical + laboratory + CT	O	[52]
LSTM	Laboratory	D	[51]
LSTM	X-ray	D	[21]
MH-CovidNet	X-ray	D	[37]
Multi-layer perceptron	Clinical	O	[30]
Multivariate logistic regression	Clinical + laboratory	O	[31]
Random forest	Clinical + laboratory	O	[61]
Random forest	Clinical + laboratory + CT	O	[62]
ResNet	CT	D	[33, 53, 54, 62, 65, 78]
Resnet	X-Ray	D	[26, 28, 66, 76]
SqueezeNet	CT	D	[75]
Support vector machine	Clinical	D	[45]
Support vector machine	Clinical + laboratory + CT	O	[29]
U- Net	Clinical + laboratory + CT	O	[52]
U-Net	CT	D	[35, 56, 59, 63, 74]
VGG	CT	D	[40]
VGG	X-Ray	D	[26, 28, 55, 57, 70, 72]
XGboost	Clinical + laboratory	O	[83]

*D* diagnosis, *O* outcome

Logistic regression (LR) is a statistical model that uses a logistic function to model a binary dependent variable that has two levels. In general, LR and other algorithms such as SVM [97] and multi-layer perceptron (MLP)[98], decision tree-based algorithms [99] and XGboost [100]) were applied to structured data.

LR was used in 6 papers that correspond of 9% of the total, and only one (1.5%) is CT Image, other is only laboratory data, 3 (5%) are clinical and laboratory data, and 1 (1.5%) is clinical, laboratory and CT image. SVM was used in 2 works (3%), with Clinical data and other with clinical, laboratory and CT image data. Moreover, as can be seen, deep learning algorithms involving architectures for images are the most frequent.

**Fig. 8:**
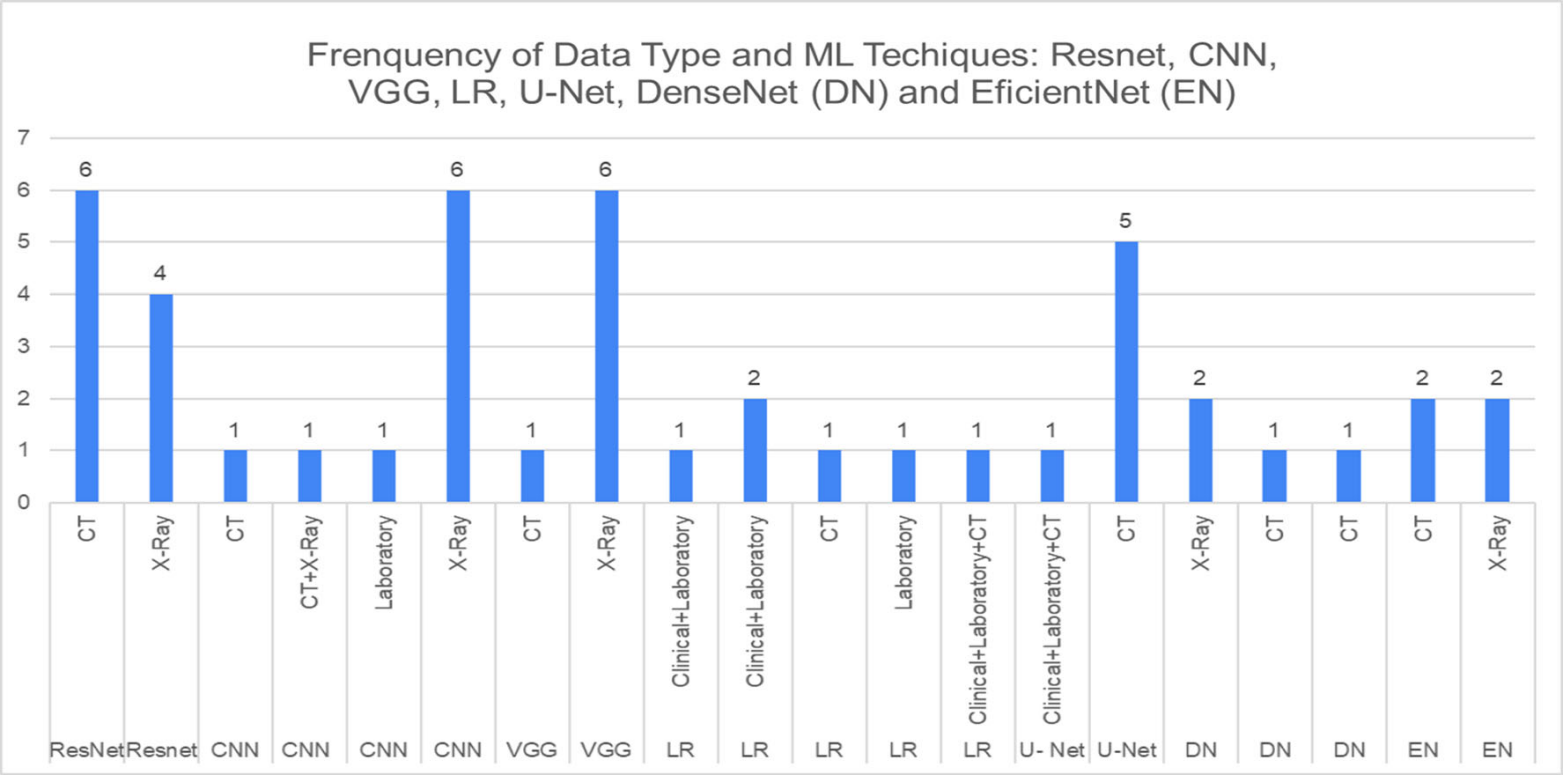
Data Types and ML techniques most used in COVID-19

Figure 8 shows an overview of the data type and the seven most used ML techniques (> 5%) according to Fig. 7: Resnet, CNN, VGG, LR, U-Net, DenseNet, and EfficientNet. We can conclude that CNN and VGG are the most applied techniques in X-ray (9,4%) and Resnet in CT (9,4%) image data. The CT is also used in U-Net (7,8%) and the X-ray in Resnet (6,3%). LR is the most used technique of structure data.

All works using Resnet, CNN, VGG and EfficientNet addressed to diagnosis, only one work of U-Net and other of DenseNet have the purpose of outcome. The LR was used in 6 papers, and only one treat Diagnosis with clinical and laboratory data, all the others 5 are about outcome (1 is Image CT data, 2 are clinical and laboratory data, 1 is clinical, laboratory and image CT data).

## Limitations

This review presents some limitations related to the selected keywords that adopted general terms, not considering specific names of IA/ML techniques. Research using AI/ML is increasing relentlessly, particularly COVID-19 context. So, a specific time was adopted, considering the period before the dissemination of vaccines, in which there was an intense search for patterns that would help in the diagnosis and outcome of the disease. Only papers with AI/ML approaches for the diagnosis or outcome of patients with COVID-19 were considered. Maybe some relevant studies were lost, but the search focused on efficient AI/ML models to identify papers with these objectives. As emphasized by Abd-Alrazaq et al. [18] the restriction of works in English may not have been considered significant works written in other languages.

## Conclusion

In this systematic literature review, we investigate AI’s main scope and contributions to the diagnosis and outcomes of COVID-19, considering the beginning of the pandemic, which was a period dedicated to the exploration of AI-based techniques aimed at identifying some patterns that mapped relationships between clinical data and images with diagnoses and outcomes. This search began by collecting 839 articles, and after going through all the steps of the selection criteria, it grouped 64 selected studies that were analyzed in this systematic review. In both contexts, medical imaging analyses, whether chest X-Ray, chest CT or ultrasound, were used, especially in the case of studies aimed at diagnosis. Among the imaging exams, the use of chest CT stands out. However, clinical and laboratory data were used more frequently in papers involving outcome.

Generally, a sensitive point for models based on DL is the quantity of data available for training the models. Specifically, for works related to semantic segmentation of medical images, there is a need for manual annotation of the images of the areas of interest in the images. This work is expense and arduous due to its high complexity and needing experienced doctors to generate an accurate annotation of the medical image. To overcome this problem, the authors use transfer learning and data-augmentation techniques to mitigate this restriction [55, 62, 66, 69, 70, 76, 77].

For structured data (clinical and laboratory data), which generally have many attributes, close to 60% of the papers used some technique to reduce the data elements by selecting or compressing the data.

It is important to emphasize that the results obtained with image processing or clinical and laboratory data are, in many cases, above 95% of accuracy, indicating that AI techniques are valuable for decision making in a more sensitive area such as healthcare. However, one of the biggest challenges in the area seems to be the lack of explanation offered by the results found by the models. In this sense, one can point out that explainable artificial intelligence [101] has been growing to fill this gap.

Combining the terms CNN, the most frequent network model in this systematic review, Covid-19 and considering the 2022 year, a quick search was performed on Scholar-Google. More than 1000 papers were listed. This fact reinforces the importance of more specific systematic reviews considering the AI/ML techniques.

This work provides medical and AI researchers with a broad overview of AI contributions involving any type of data to the development of research to combat COVID-19, aiming to inspire new work to continue maximizing the advantages of AI with small or large databases to combat this pandemic. Finally, it is important to stress that in the initial of the disease was thought to affect mainly the lungs. Today it is known that it is a systemic disease that affects other organs and physiological systems [102]. In this context, new challenges arise for the automation of diagnoses and outcomes, integrating, in addition to images, clinical, and laboratory data. Although most patients recovered, several long-lasting symptoms were described and named “long-term COVID-19 syndrome” [103]. In this case, AI/ML models can be exploited from patient data to identify the potential to develop such a syndrome early and provide rehabilitation support more quickly. Other topics that can be further explored from an AI point of view are related to the diagnosis of Covid-19 in pediatric patients and the prediction of the duration of hospitalization in COVID-19 patients, considering ML models with a high level of accuracy [104].

## Data Availability

The data and materials used can be found in the references of this work.

## Abbreviations

AD3D0MIL: Attention-based deep 3D multiple instance learning
AFS-DF: Adaptive feature selection guided deep forest
AI: Artificial intelligence
AlexNet: Is the name of a convolutional neural network (CNN) architecture, designed by Alex Krizhevsky
ANN: Artificial neural network
AUC: Area under the ROC curve
CNN: Convolution neural networks
CNNLSTM: Convolutional neural network long-short time memory
CNNRNN: Convolutional neural networks recurrent neural networks
ConvLSTM: Convolutional long short-term memory
COVID-19: Coronavirus disease 2019
CT: Computed tomography
DeepLabv3: The third version of the system of semantic image segmentation task
DenseNet121: Densely connected CNN with 121 layers, an architecture of convolutional neural network
DenseNet201: Densely connected CNN with 201 layers, an of convolutional neural network
DL: Deep learning
DNA: Deoxyribonucleic acid (abbreviated)
EfficientNet: Are a family of models based on deep learning scaled up very effectively
GAN: Generative adversarial network
Grad-CAM: Gradient-weighted class activation mapping
H1n1: Flu that is known popularly as Swine flu
ImageNet: **T** is an image database organized according to the WordNet hierarchy (currently only the nouns), in which each node of the hierarchy is depicted by hundreds and thousands of images
InceptionV3: Is a convolutional neural network for assisting in image analysis and object detection
KNN: K-nearest neighbor
LR: Logist regression
LSTM: Long-short time memory
MERS: Middle east respiratory syndrome
MH-CovidNet: Is the name given by its authors to the proposed methodology that joins deep learning algorithms and meta-heuristic algorithms
ML: Machine learning
nCOVNet: Deep learning neural network-based method
PRISMA: Preferred reporting items for systematic reviews and meta-analyses
ReLu: Rectified linear unit activation function
ResNet: Residual neural network
ResNet50: Is a convolutional neural network that is 50 layers deep
RF: Decision tree-based algorithms
RNA: Ribonucleic acid (abbreviated)
RNN: Recurrent neural networks
ROC: Receiver operating characteristic
SARS: Severe acute respiratory syndrome
SARS-CoV-2: Severe acute respiratory syndrome coronavirus 2
SVM: Decision tree, support vector machine
U-Net: Is a convolutional neural network that was developed for biomedical image segmentation
VGG: Is a classical convolutional neural network architecture, stands for visual geometry group
VGG-16: Is a convolutional neural network model with 16 layers
WHO: World Health Organization
XGboost: Method that seeks to improve the performance of gradient boosting by optimizing

## Footnotes

1 https://covid19.who.int/.

2 https://www.mendeley.com/guides.

3 https://www.cdc.gov/coronavirus/2019-ncov/lab/virus-requests.html.
